# Trends and Disparities in Mortality From Pneumonia and Cerebrovascular Disease in the United States (1999–2020)

**DOI:** 10.1002/brb3.71849

**Published:** 2026-10-22

**Authors:** Muhammad Abdullah, Masab Ali, Taha Mehmood, Muhammad Hassan, Aasim Ali, Amina Jamil, Muhammad Umar Shahid, Areej Asif, Aliha Wahab, Muhammad Husnain Ahmad, Noor Anwar, Hassaan Ahmed Bhatti

**Affiliations:** ^1^ Department of Internal Medicine Faisalabad Medical University Faisalabad Punjab Pakistan; ^2^ Department of Neurology Allied Hospital Faisalabad Pakistan; ^3^ Department of Internal Medicine Aziz Fatima Medical and Dental College Faisalabad Pakistan; ^4^ Fatima Jinnah Medical University Lahore Pakistan; ^5^ Department of Internal Medicine Ghurki Trust Hospital Lahore Punjab Pakistan; ^6^ Department of Medicine Tentishev Satkynbai Memorial Asian Medical Institute Kant Kyrgyzstan; ^7^ Independent Medical College Faisalabad Pakistan; ^8^ Fatima Memorial Hospital Lahore Pakistan

**Keywords:** cerebrovascular disease, disparities, joinpoint regression, mortality, pneumonia, stroke‐associated pneumonia

## Abstract

**Background:**

Pneumonia is a frequent and often fatal complication of cerebrovascular disease (CVD). Stroke care has improved considerably since the late 1990s, but national trends in deaths involving both conditions and their distribution across demographic and geographic groups have not been described.

**Methods:**

We used the Centers for Disease Control and Prevention Wide Ranging Online Data for Epidemiologic Research (CDC WONDER) Multiple Cause of Death files to identify United States decedents aged 55 years and older who died between 1999 and 2020 with pneumonia (ICD‐10 codes J18.0, J18.1, J18.2, J18.8, and J18.9) and CVD (I60–I69), both recorded among the causes of death. Age‐adjusted mortality rates (AAMRs) per 100,000 populations were standardized to the 2000 United States population. Joinpoint regression estimated the annual percent change (APC) and average annual percent change (AAPC) overall and by sex, age group, race and ethnicity, urbanization, and census region. A sensitivity analysis of the overall series used a broader pneumonia definition (J12–J18).

**Results:**

We identified 316,760 deaths involving both conditions. The overall AAMR fell from 45.4 per 100,000 in 1999 to 9.1 in 2019 (80.0% decrease) and rose to 15.8 in 2020 (73.6% above the 2019 rate); the AAPC for 1999 to 2020 was −5.09% (95% confidence interval [CI] −5.92 to −4.26). Every stratum showed a net decline over the full period and an increase in 2020. Rates were higher throughout in men, in adults aged 75 years, and older and in nonmetropolitan counties and were highest in non‐Hispanic Black adults in every year except 2017. Absolute differences between groups narrowed as overall rates fell. The relative increase in 2020 was largest in non‐Hispanic American Indian or Alaska Native (160%), Hispanic (144%), and non‐Hispanic Black (115%) adults and in adults aged 55–74 years (97%). The broader pneumonia definition added 15,521 deaths (4.9%) and produced the same joinpoints and a similar AAPC (−4.89%).

**Conclusion:**

Deaths involving pneumonia and CVD fell by about 80% between 1999 and 2019 and rose sharply in 2020, coinciding with the COVID‐19 pandemic. Disparities by sex, age, race and ethnicity and urbanization persisted, and the 2020 increase was proportionally larger in several minority groups. These aggregate certificate data describe patterns but cannot identify their causes. Studies with individual‐level clinical data are needed to test whether dysphagia screening, vaccination, and infection prevention explain the long‐term decline and the disparities.

## Graphical abstract

Graphical abstract summarizing trends in deaths involving both pneumonia and cerebrovascular disease among US adults aged 55 years and older, 1999 to 2020. Panel A: the study used national death certificate records and identified 316,760 deaths on which both pneumonia and cerebrovascular disease, including stroke, were listed among the causes of death. Panel B: age adjusted death rates per 100,000 people were compared by sex, age group, race and ethnicity, urbanization and census region, and trends were tested with joinpoint regression, which identified turning points in 2003, 2009 and 2018. A broader pneumonia definition gave the same pattern. The trend line is a schematic and is not to scale. Panel C: the age adjusted death rate fell from 45.4 per 100,000 in 1999 to 9.1 in 2019, a decline of about 80%, then rose to 15.8 in 2020, an increase of 74% over 2019, the first year of the COVID pandemic. The 2020 rise was largest in American Indian or Alaska Native, Hispanic and Black adults.

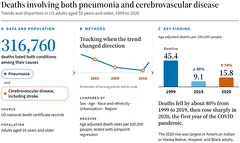

**Abbreviations**
AAMR: age‐adjusted mortality rate
AAPC: average annual percent change
APC: annual percent change
CDC WONDER: Centers for Disease Control and Prevention Wide Ranging Online Data for Epidemiologic Research
CI: confidence interval
CVD: cerebrovascular disease
ICD‐10: International Classification of Diseases Tenth Revision
MCD: Multiple Cause of Death
NCHS: National Center for Health Statistics

## 1 Introduction

Pneumonia is a common cause of morbidity and mortality worldwide, especially in the elderly and in individuals with chronic comorbid conditions. According to the World Health Organization, lower respiratory infections are among the leading causes of death globally and contribute substantially to the burden of disease in older populations (World Health Organization 2024). In the United States, pneumonia remains an important cause of death, particularly in vulnerable groups such as those living with neurological impairment (Centers for Disease Control and Prevention).

Cerebrovascular disease (CVD), including ischemic and hemorrhagic stroke, is a leading contributor to long‐term disability and death in the United States. Stroke survivors are predisposed to pneumonia through several converging mechanisms, including impaired swallowing and cough reflexes, reduced level of consciousness, immobility, and stroke‐induced immunodepression (Westendorp et al. 2011; Iadecola and Anrather 2011). Pneumonia arising in this setting is among the most frequent complications of acute cerebrovascular events and is associated with longer hospital stay, poorer functional recovery, and higher case fatality (Hannawi et al. 2013).

Despite advances in stroke management, including early reperfusion therapy and better organized supportive care, poststroke infection remains a major clinical problem. Infection, particularly pneumonia, accounts for a considerable share of deaths that are in principle preventable (Westendorp et al. 2011; Hannawi et al. 2013). Reported outcomes also differ across population groups, with a greater burden described among older adults, men, and several racial and ethnic minority populations (Benjamin et al. 2019).

National vital statistics allow long‐term mortality patterns to be examined at the population level and report from the National Center for Health Statistics (NCHS) document sizeable age‐adjusted declines for several leading causes of death, alongside evidence that some gains stalled or reversed in recent years (Centers for Disease Control and Prevention).

What remains poorly characterized is the intersection of these two conditions. Most published work on pneumonia after stroke comes from single centers, registries, or short observation windows and cannot describe national trends or differences between demographic and geographic groups. The effect of the health system disruption of 2020 on this population has not been quantified.

This descriptive surveillance study examines national trends in mortality involving both pneumonia and CVD among United States adults aged 55 years and older between 1999 and 2020, using the Centers for Disease Control and Prevention Wide Ranging Online Data for Epidemiologic Research (CDC WONDER) Multiple Cause of Death (MCD) files. We describe temporal patterns overall and by sex, age group, race and ethnicity, urbanization, census region, state, and place of death, and we quantify the change in 2020 relative to the preceding decline. The aim is to show where the burden is concentrated, not to identify its causes.

## 2 Methods

### 2.1 Study Design and Data Source

This was a retrospective, serial cross‐sectional analysis of national death certificate data. Mortality records were obtained from the MCD component of the CDC WONDER platform, which compiles information from all death certificates filed in the 50 states and the District of Columbia and is maintained by the NCHS (Centers for Disease Control and Prevention 2024; CDC 2024). Each record in this file contains one underlying cause of death and up to 20 additional conditions listed anywhere on the certificate. Comparable serial cross‐sectional designs using this data source have been published previously (Nazir et al. 2022; Jain et al. 2022; Ali et al. 2026), and the present analysis follows that established approach.

Because CDC WONDER captures the complete national census of registered deaths rather than a sample, no sample size calculation was required, and precision is limited only by the number of events within each subgroup. The study used de‐identified, publicly available data and therefore did not constitute human subjects research and required no institutional review board approval. Reporting follows the Strengthening the Reporting of Observational Studies in Epidemiology (STROBE) statement for cross‐sectional studies (Cuschieri 2019).

### 2.2 Case Definition and Query Specification

The exact query is specified so that the extraction can be reproduced. Within the MCD ICD‐10 code field, pneumonia was selected using J18.0 (bronchopneumonia, unspecified), J18.1 (lobar pneumonia, unspecified), J18.2 (hypostatic pneumonia, unspecified), J18.8 (other pneumonia, organism unspecified), and J18.9 (pneumonia, unspecified). These were combined, using the AND operator in the MCD query interface, with the CVD block I60–I69 (World Health Organization 2019), also selected within the MCD field. A death entered the analysis only if both a J18 code and an I60–I69 code appeared among the conditions recorded on the certificate, irrespective of which was the underlying cause. No restriction was placed on the underlying cause field, because either condition may plausibly be certified as the underlying cause in this population.

The J18 group was chosen as the primary definition because it captures pneumonia certified without a specified organism and includes the bronchopneumonia and hypostatic pneumonia codes; pneumonia ascertainment is known to be heterogeneous in stroke populations (Kishore et al. 2015). Codes for pneumonia with a specified organism (J12–J17), influenza (J09–J11), and aspiration pneumonitis (J69.0) were not part of the primary definition. Because omitting J12–J17 could affect the outcome, a broader definition was evaluated in a sensitivity analysis. Aspiration pneumonitis and influenza codes were not evaluated and are addressed in Section 4.1.

### 2.3 Sensitivity Analysis

To assess whether the findings depended on the pneumonia definition, the overall analysis was repeated with pneumonia defined as ICD‐10 J12–J18, combined with I60–I69 using the same query structure, age range, age adjustment, and joinpoint settings. This sensitivity analysis was limited to the overall series. We compared the number of deaths, age‐adjusted mortality rates (AAMRs) in 1999, 2019, and 2020, joinpoint years, annual percent changes (APCs), and the average annual percent change (AAPC) between the two definitions and calculated for each year the number of deaths added by the broader definition (deaths with a J12–J17 code and no J18 code) and their share of all deaths under the broader definition.

### 2.4 Study Population and Demographic Variables

The analysis was restricted to decedents aged 55 years and older, because deaths involving both conditions at younger ages were too few to yield stable or publishable rates under NCHS reliability and confidentiality rules. The 10‐year age groups available in CDC WONDER (55–64, 65–74, 75–84, and 85 years and older) were collapsed into 55–74 years and 75 years and older for stratified analysis, which preserved enough events for joinpoint estimation in each stratum.

Race and ethnicity were derived from the race and Hispanic origin fields on the death certificate according to Office of Management and Budget standards as implemented by NCHS (CDC 2024). Because CDC WONDER holds these as separate variables, two extractions were combined: Hispanic or Latino (any race) and, among decedents not Hispanic or Latino, the bridged race categories. This produced five mutually exclusive groups: Hispanic or Latino, non‐Hispanic American Indian or Alaska Native, non‐Hispanic Asian or Pacific Islander, non‐Hispanic Black or African American, and non‐Hispanic White. Deaths with Hispanic origin not stated were excluded from this stratification only. Sex was recorded as male or female. Place of death was classified as medical facility, nursing home or long‐term care facility, decedent's home, hospice facility, or other and unknown locations.

Geographic analyses used the four United States Census Bureau regions (Northeast, Midwest, South, and West) and state of residence. Urbanization was assigned using the NCHS Urban Rural Classification Scheme for Counties (CDC 2024; Ingram and Franco 2013); the four metropolitan categories were combined as metropolitan and the micropolitan and noncore categories as nonmetropolitan. Records with missing or unclassified demographic or geographic information were omitted from the relevant subgroup analysis but retained in overall counts; they accounted for less than 1% of records.

### 2.5 Statistical Analysis

AAMRs per 100,000 populations were obtained from CDC WONDER by the direct method using the 2000 United States standard population (Arias et al. 2021). Rates based on fewer than 20 deaths are flagged as unreliable by NCHS and were not interpreted. Percent changes between years were calculated from the published rates.

Trends were modeled with the Joinpoint Regression Program, version 5.0.2 (National Cancer Institute) (Joinpoint Regression Program), fitting log‐linear models to the annual AAMR series. Given 22 annual observations, we permitted a maximum of four joinpoints, with the number selected by the permutation test, using the grid search method with a (2,2,0) specification. The APC for each segment and the AAPC across 1999–2020 were estimated with 95% confidence intervals (CIs) as produced by the program and were considered statistically significant when *p* was less than 0.05. A final segment that begins at the penultimate observation, such as 2018–2020, rests on three annual values, and its estimate should be interpreted with caution.

 *p* values are reported to three decimal places, with values below 0.001 reported as *p* less than 0.001. Because the analysis was limited to deaths coded under ICD‐10, which has been used for United States mortality data since 1999, no bridging across classification versions was required. All rates relate to deaths in which both conditions were recorded and use the general population aged 55 years and older as the denominator; they do not represent case fatality among people with CVD.

## 3 Results

Between 1999 and 2020, 316,760 deaths in adults aged 55 years and older listed both pneumonia and CVD among the causes of death. Table S12 summarizes AAMRs, percent changes and AAPCs for every stratum; the full joinpoint estimates are in Table S3 and the annual series in Tables S1–S9.

### 3.1 Overall Trend and Place of Death

The AAMR fell from 45.4 per 100,000 in 1999 to 9.1 in 2019 (80.0% reduction) and rose to 15.8 in 2020, 73.6% above the 2019 rate (Figure 1). Joinpoint regression identified three joinpoints (2003, 2009, and 2018). The APC was −6.48% during 1999–2003 (not significant), −11.61% during 2003–2009, and −6.41% during 2009–2018, followed by an increase of 28.85% per year during 2018–2020 (95% CI 14.78–39.73; *p* less than 0.001). The AAPC over the full period was −5.09% (95% CI −5.92 to −4.26; *p* less than 0.001). The 2018–2020 segment rests on three annual values and mainly reflects 2020, because the 2019 rate (9.1) was slightly lower than the 2018 rate (9.6). The 2020 rate remained below the 1999 rate in every group, and every stratum in Table S12 showed the same shape: a decline through 2018 or 2019 and an increase in 2020.

**FIGURE 1:**
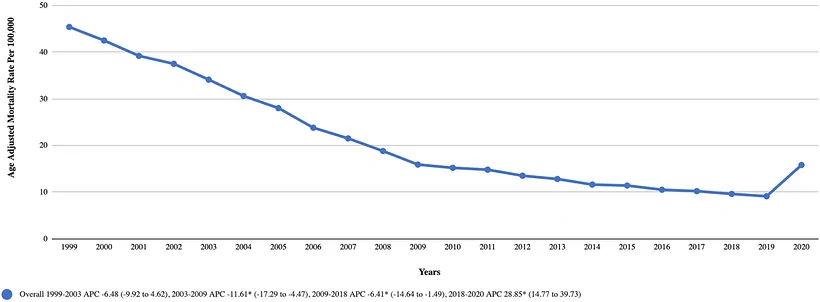
Overall pneumonia and cerebrovascular disease‐related age‐adjusted mortality rates per 100,000 in the United States, 1999–2020. * indicates that the annual percentage change (APC) is significantly different from zero at *α* = 0.05. AAMR = age‐adjusted mortality rate.

Most deaths occurred in medical facilities (175,597; 55.4%) or in nursing homes and long‐term care facilities (104,185; 32.9%), followed by the decedent's home (21,349; 6.7%), hospice facilities (8720; 2.8%), and other or unspecified locations (6909; 2.2%). The nursing home share fell from 39.4% in 1999 to 22.3% in 2019 and 19.3% in 2020, whereas the medical facility share rose from 56.1% in 2019 to 62.3% in 2020 (Tables S2 and S12). Hospice deaths are separately identifiable only from 2003.

### 3.2 Sex

Men had higher AAMRs than women in every year (Figure 2), although women accounted for more deaths (163,018 vs. 153,742). The male to female rate ratio was 1.54 in 1999, 1.59 in 2019, and 1.73 in 2020. The relative increase in 2020 was larger in men (79.3%) than in women (64.4%). In women, the 2018–2020 increase was not statistically significant (APC 23.62%; 95% CI −3.65 to 35.83; *p* = 0.088).

**FIGURE 2:**
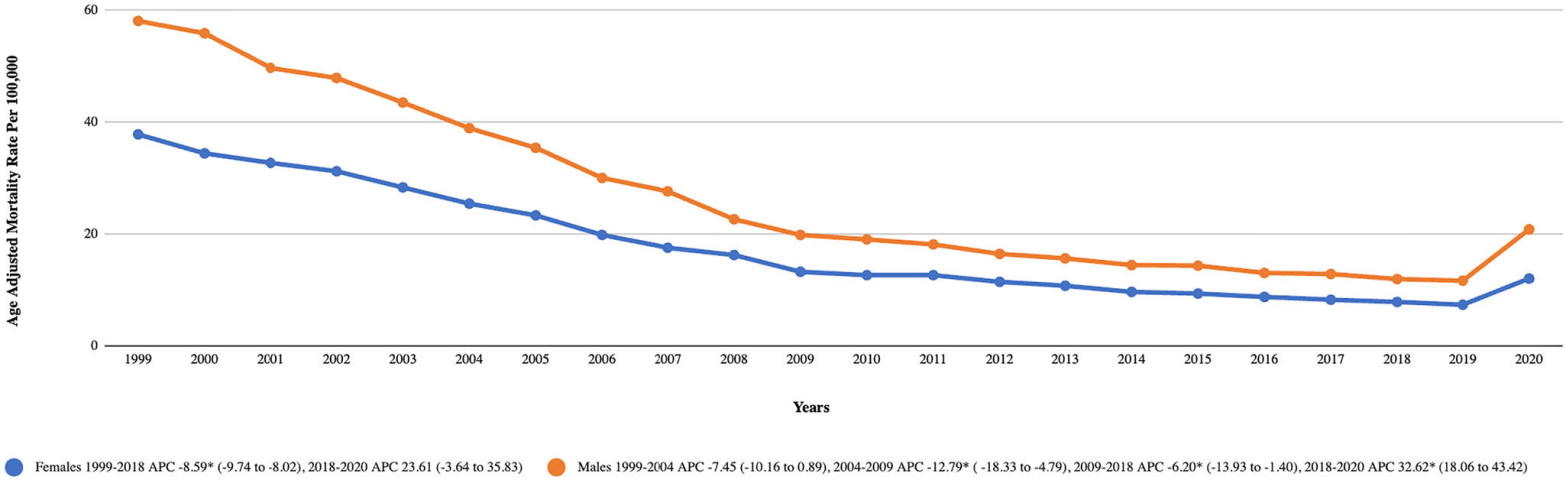
Pneumonia and cerebrovascular disease‐related age‐adjusted mortality rates per 100,000 stratified by *gender* in the United States, 1999–2020. * indicates that the annual percentage change (APC) is significantly different from zero at *α* = 0.05. AAMR = age‐adjusted mortality rate.

### 3.3 Age Group

AAMRs were higher in adults aged 75 years and older than in those aged 55–74 years in every year (Figure 3). The ratio between the groups narrowed from 11.7 in 1999 to 6.7 in 2019, and the absolute gap from 120.7 to 20.0 per 100,000. The younger group declined less (69.0% vs. 82.2%; AAPC −2.85% vs. −6.03%), and its rate changed little after 2012 (3.9 in 2012 and 3.5 in 2019). In 2020 the relative increase was larger in adults aged 55–74 years (97.1%) than in those aged 75 years and older (63.4%), although the absolute increase was smaller (3.4 vs. 14.9 per 100,000).

**FIGURE 3:**
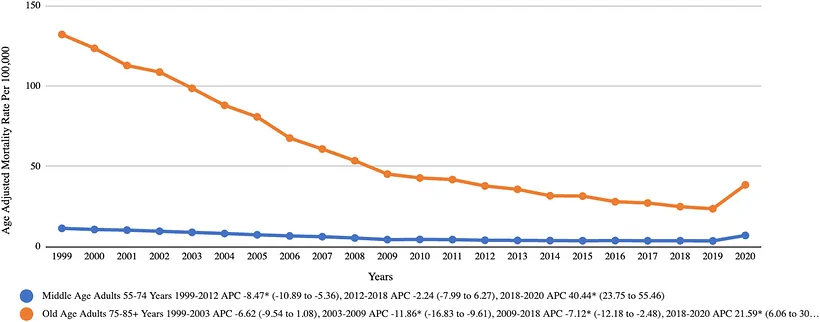
Pneumonia and cerebrovascular disease‐related age‐adjusted mortality rates per 100,000 stratified by *age* in the United States, 1999–2020. * indicates that the annual percentage change (APC) is significantly different from zero at *α* = 0.05. AAMR = age‐adjusted mortality rate.

### 3.4 Race and Ethnicity

AAMRs fell by 77.2%–82.5% in all five groups between 1999 and 2019 (Figure 4). Non‐Hispanic Black adults had the highest rate in every year except 2017, when the rate in non‐Hispanic American Indian or Alaska Native adults was higher (17.4 vs. 15.3). The absolute spread across groups narrowed from 19.3 per 100,000 in 1999 (range 40.7–60.0) to 5.5 in 2019 (range 8.2–13.7), whereas the ratio of the non‐Hispanic Black to the non‐Hispanic White rate rose from 1.37 to 1.59. In 2020, the spread widened to 16.3 per 100,000 (range 13.2–29.5), and the ratio to 2.23. The relative increase in 2020 was largest in non‐Hispanic American Indian or Alaska Native (159.8%), Hispanic or Latino (144.2%), and non‐Hispanic Black (115.3%) adults, compared with 84.4% in non‐Hispanic Asian or Pacific Islander and 53.5% in non‐Hispanic White adults. The American Indian or Alaska Native series was irregular, with year‐to‐year reversals, the widest CIs of any group, and a nonsignificant 2018–2020 increase (APC 37.00%; 95% CI −2.42 to 66.16; *p* = 0.080).

**FIGURE 4:**
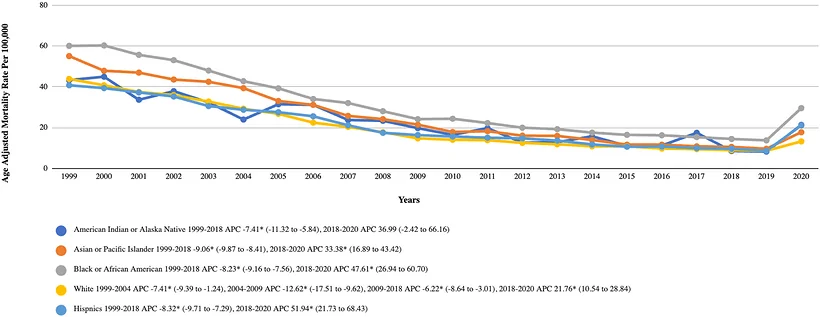
Pneumonia and cerebrovascular disease‐related age‐adjusted mortality rates per 100,000 stratified by *race* in the United States, 1999–2020. * indicates that the annual percentage change (APC) is significantly different from zero at *α* = 0.05. AAMR = age‐adjusted mortality rate.

### 3.5 Urbanization

AAMRs were higher in nonmetropolitan than in metropolitan counties in every year (Figure 5). The absolute difference narrowed from 6.1 per 100,000 in 1999 to 1.7 in 2019 and was 1.6 in 2020, whereas the rate ratio changed little (1.14 in 1999, 1.19 in 2019, and 1.10 in 2020). The relative increase in 2020 was larger in metropolitan areas (74.2%) than in nonmetropolitan counties (61.3%).

**FIGURE 5:**
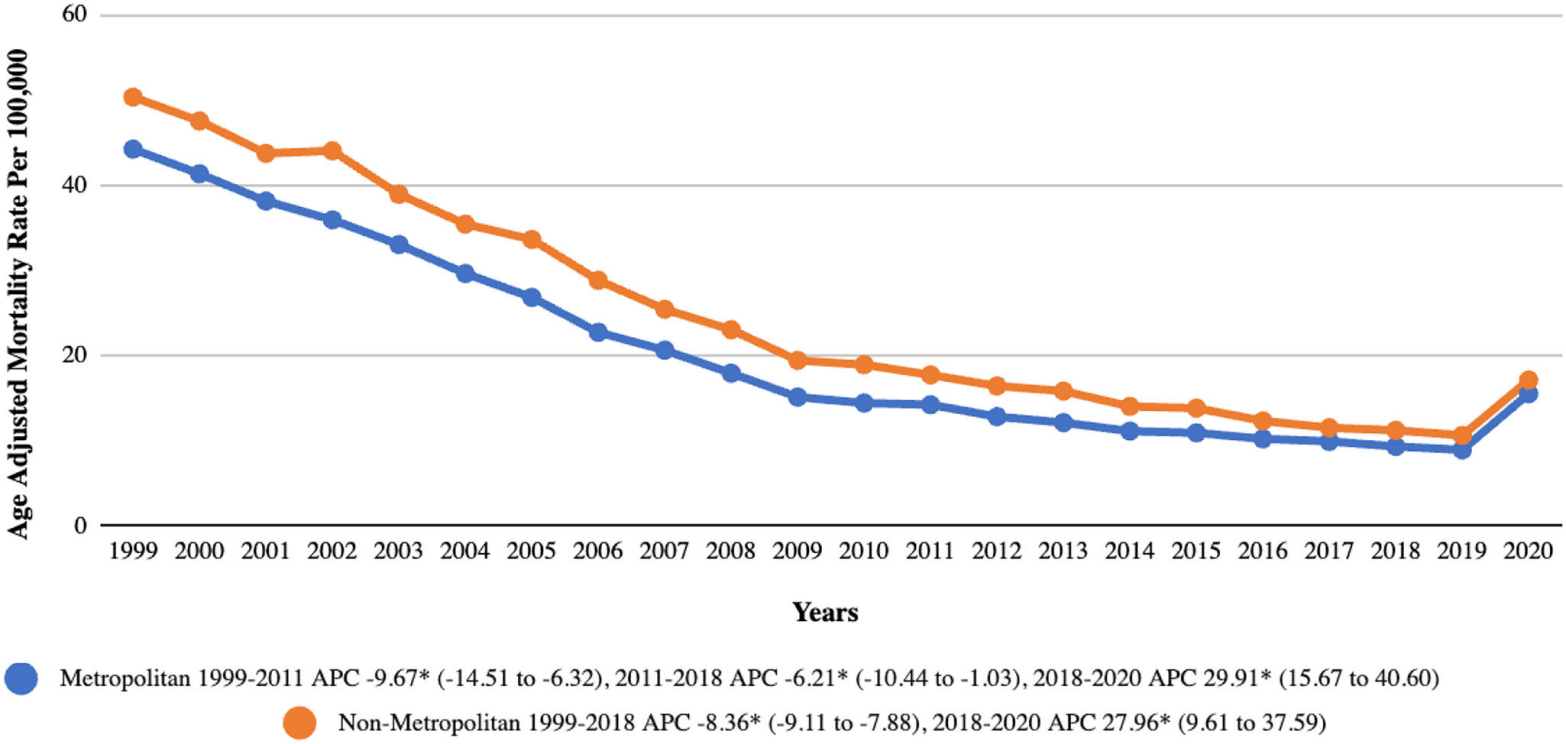
Pneumonia and cerebrovascular disease‐related age‐adjusted mortality rates per 100,000 stratified by *urban–rural* in the United States, 1999–2020. * indicates that the annual percentage change (APC) is significantly different from zero at *α* = 0.05. AAMR = age‐adjusted mortality rate.

### 3.6 Census Region

AAMRs fell in all four regions (Figure 6). The West began with the highest rate in 1999 (55.7) and showed the steepest decline (84.0%; AAPC −6.61%), so that the four regional rates in 2019 (8.6–9.4) lay within 0.8 per 100,000 of one another, compared with a spread of 18.5 in 1999. In 2020, the South had the highest rate (17.1) and the largest relative increase (81.9%), and the West the smallest (61.8%).

**FIGURE 6:**
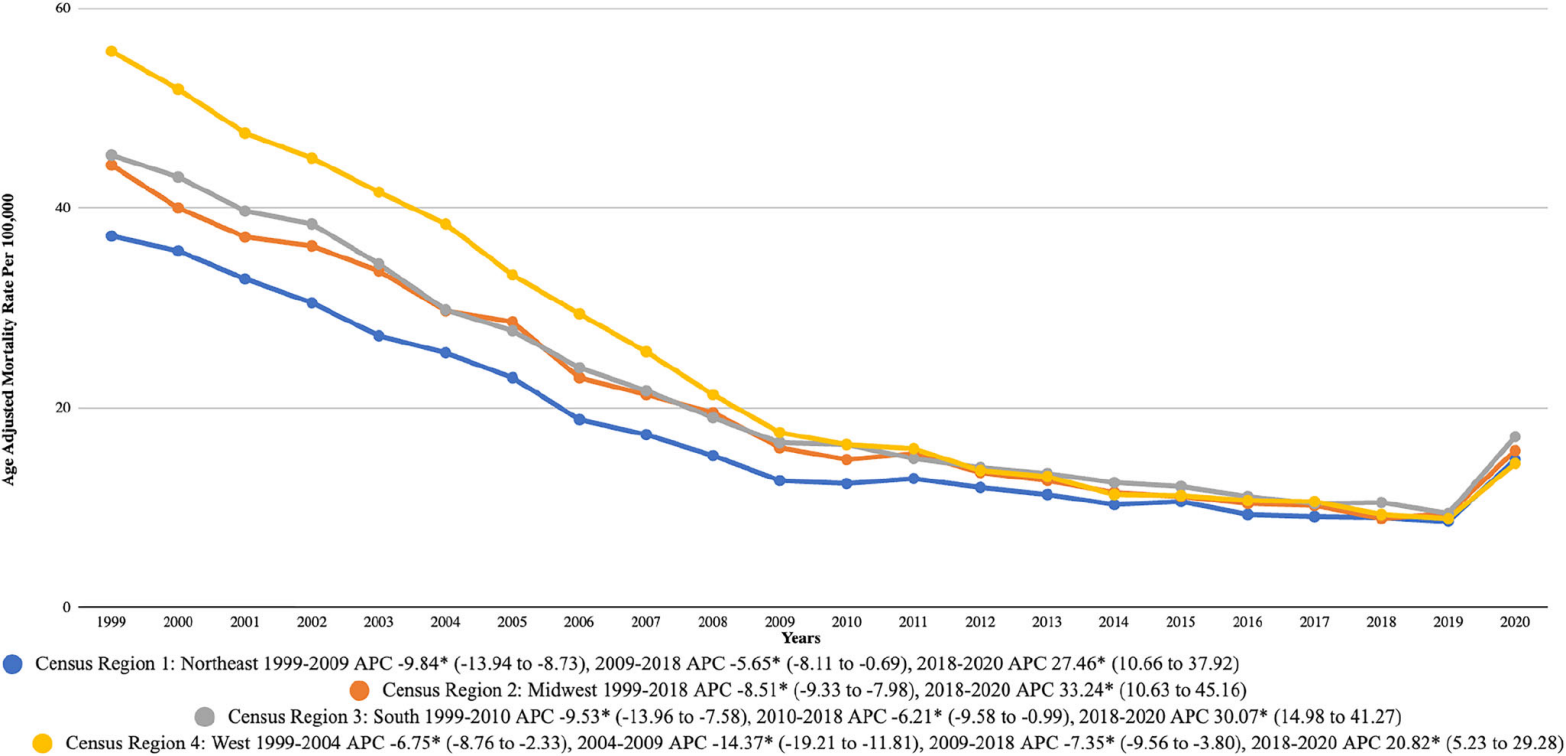
Pneumonia and cerebrovascular disease‐related age‐adjusted mortality rates per 100,000 stratified by *consensus region* in the United States, 1999–2020. * indicates that the annual percentage change (APC) is significantly different from zero at *α* = 0.05. AAMR = age‐adjusted mortality rate.

### 3.7 State

Pooled state AAMRs for 1999–2020 ranged from 9.6 to 28.4 per 100,000 (Table S7). The highest were in West Virginia (28.4), California (28.1), and Oklahoma (27.1), and the lowest were in Arizona (9.6), Florida (10.9), and Nevada (11.9). Pooled rates are weighted toward the early years, when rates were highest, and should not be compared with single‐year rates; the high pooled rate in California is consistent with the high regional rate in the West in 1999. California contributed the most deaths (47,374; 15.0%), followed by Texas (21,628; 6.8%).

### 3.8 Sensitivity Analysis Using a Broader Pneumonia Definition

With pneumonia defined as J12–J18, 332,281 deaths were identified, 15,521 (4.9%) more than under the primary definition, so the primary definition captured 95.3% of deaths under the broader definition. The added deaths were spread across all years and accounted for 3.2% of deaths under the broader definition in 1999, 8.2% in 2019, and 9.3% in 2020 (Table S10). The AAMR fell from 46.90 per 100,000 in 1999 to 9.96 in 2019 (78.8% reduction) and rose to 17.43 in 2020 (75.0% increase). Joinpoint regression identified the same three joinpoints (2003, 2009, and 2018), and the 2018–2020 APC was 29.08% (95% CI 12.58–39.01) with an AAPC of −4.89% (95% CI −5.86 to −4.21), compared with −5.09% under the primary definition (Tables S1 and S13). The direction and timing of the trends were therefore not sensitive to the definition; the slightly smaller decline is consistent with a modest rise over time in the share of deaths carrying an organism‐specific pneumonia code. This analysis was limited to the overall series.

## 4 Discussion

In this national analysis of death certificate data, mortality involving both pneumonia and CVD among adults aged 55 years and older fell by about 80% between 1999 and 2019 and then rose by 73.6% in 2020, and every sex, age, race and ethnicity, urbanization, and regional stratum followed the same overall pattern. A broader pneumonia definition gave the same joinpoints and a similar AAPC, so the pattern does not appear to depend on the code set used. These are descriptive findings from aggregate certificate data, and the explanations offered below are hypotheses rather than results.

For context, national data for adults aged 65 years and older show that the age‐adjusted stroke death rate (underlying cause, I60–I69) fell by about 41% between 2000 and 2019 and rose by about 4% in 2020 (QuickStats 2022). Our series fell roughly twice as steeply and rose far more in 2020. The two series differ in age floor and cause field and are not directly comparable, but the contrast suggests that deaths with pneumonia recorded changed more than stroke deaths overall, whether through changes in pneumonia occurrence or in how it is recorded.

The long‐term decline may partly reflect improvements in stroke care and pneumonia prevention, such as organized stroke unit care (Langhorne et al. 2013; Westendorp et al. 2011), dysphagia screening, oral care and early mobilization, and higher pneumococcal and influenza vaccination coverage in older adults (Matanock et al. 2019). None of these were measured here. Changes in stroke incidence and case fatality, in the setting of death (the nursing home share of these deaths fell from 39.4% to 22.3% between 1999 and 2019) and in certification and coding practice could also contribute. A decline of this size should not be attributed to any single factor.

The rise in 2020 coincided with the COVID‐19 pandemic. Rates were still falling in 2019, so the upturn is a feature of 2020 alone. CVD has been associated with worse outcomes in COVID‐19 (Fraiman et al. 2020; Nannoni et al. 2021), and disruption of care may also have contributed, but neither COVID‐19 status nor care disruption was measured. Some deaths certified with pneumonia in 2020 may be COVID‐19 pneumonia, particularly early in the pandemic when testing was limited, so the increase reflects a combination of possible excess pneumonia mortality and changed certification that these data cannot separate. Place of death shifted toward medical facilities in 2020 (62.3% vs. 56.1% in 2019), and long‐term care facilities were sites of severe early outbreaks (McMichael et al. 2020), but the data do not show why the shift occurred. Analyses that use COVID‐19 codes (U07.1) recorded on the same certificates would help to separate these components.

Rates were higher in men, in adults aged 75 years and older, in nonmetropolitan counties, and, in all years but one, in non‐Hispanic Black adults. These patterns are in the direction of published differences by sex (Zhao et al. 2015), age (Smith et al. 2015; Finlayson et al. 2011), race (Cushman 2025; Skolarus et al. 2020), and rural residence (Cross et al. 2021), but the data contain no information on comorbidity, stroke severity, dysphagia, insurance, or quality of care, so the reasons cannot be determined. The relative gap between men and women (rate ratio 1.54 in 1999, 1.59 in 2019) and between nonmetropolitan and metropolitan counties (1.14 and 1.19) was stable, the age ratio narrowed from 11.7 to 6.7, and the ratio of the non‐Hispanic Black to the non‐Hispanic White rate widened from 1.37 to 1.59. The wide variation between states (28.1 in California and 9.6 in Arizona, for example) may partly reflect differences in certification and coding practice rather than in disease burden and was not examined further.

Absolute differences between groups narrowed as overall rates fell; for example, the spread across racial and ethnic groups decreased from 19.3 per 100,000 in 1999 to 5.5 in 2019. Because ratios between groups did not fall, this narrowing mainly reflects the lower baseline rather than a relative improvement in higher rate groups. The 2020 increase reopened part of the racial and ethnic gap (spread 16.3 per 100,000), with the largest relative increases in American Indian or Alaska Native, Hispanic, and Black adults. The 2020 increase was not concentrated in the highest baseline groups in every comparison: It was proportionally larger in adults aged 55–74 years than in older adults and in metropolitan than in nonmetropolitan counties. The American Indian or Alaska Native series needs particular caution. It is the smallest, its annual rates were irregular, its CIs were the widest, and race is more often misclassified on death certificates for this group, which biases rates downward unevenly across states and years (Arias et al. 2016). Its pattern, including its very large relative rise in 2020 from a small base, is consistent with statistical instability and differential ascertainment and should not be read as a distinct temporal course unless confirmed with linked race data.

These findings raise questions that the present design cannot answer. If the long‐term decline reflects better prevention and care, the 2020 reversal might indicate that these gains depend on services that were disrupted during the pandemic, but this cannot be tested with aggregate data. Studies that link individual‐level data, such as stroke registries or electronic health records, to outcomes are needed to determine which components of poststroke care, if any, account for the decline, the residual disparities, and the 2020 increase, and extending the series beyond 2020 will show whether the increase was transient. Protecting dysphagia screening, oral care, and vaccination during health system emergencies is a reasonable precaution, but it is not a conclusion that follows from these data.

Strengths of this study are the complete national census of registered deaths over 22 years, joinpoint methods that locate changes in trend, and a fully specified query that allows exact reproduction.

### 4.1 Limitations

This study has several limitations. First, death certificate data are subject to misclassification and to variable completeness in the recording of contributing conditions, and both diagnoses depend on certifier practice, which may vary by state, setting, and year. Second, deaths were identified from the multiple cause field, so the sequence of events cannot be determined; the deaths described involve both conditions and are not necessarily deaths caused by pneumonia in stroke survivors. Third, rates use the general population aged 55 years and older as the denominator, so changes may reflect changes in stroke incidence, prevalence, or survival as well as changes in pneumonia risk among stroke survivors. Fourth, the primary case definition excludes pneumonia coded to a specified organism (J12–J17), influenza (J09–J11), and aspiration pneumonitis (J69.0). The sensitivity analysis with J12–J18 changed the overall results little, but it was limited to the overall series, and aspiration pneumonitis, a principal mechanism of pneumonia after stroke, was not assessed, so the burden may be underestimated by an unknown amount. Fifth, COVID‐19 codes were not examined, so the 2020 increase cannot be divided into excess pneumonia mortality and changes in certification. Sixth, the data contain no clinical information on stroke subtype and severity, dysphagia, functional dependency, comorbidity, vaccination, or socioeconomic position, so adjustment for confounding and causal inference was not possible, and community‐acquired, hospital‐acquired, and aspiration pneumonia could not be distinguished. Seventh, race and ethnicity on death certificates are misclassified, most notably for American Indian or Alaska Native and Asian or Pacific Islander decedents (Arias et al. 2016), and rates for small subgroups are unstable. Finally, the 2018–2020 joinpoint segment rests on three annual values; deaths below age 55 were not analyzed, and the series ends in 2020, so it cannot show whether the increase persisted.

## 5 Conclusion

Mortality involving both pneumonia and CVD among United States adults aged 55 years and older fell by about 80% between 1999 and 2019 and rose by 73.6% in 2020, coinciding with the COVID‐19 pandemic and reversing part of the earlier decline. Disparities by sex, age, race, and ethnicity and urbanization persisted throughout, and the relative increase in 2020 was largest in several minority groups. These aggregate data describe patterns but cannot establish their causes; individual‐level studies and surveillance beyond 2020 are needed to determine which factors account for the decline, the disparities, and the 2020 reversal.

## Author Contributions

**Muhammad Abdullah**: writing – original draft, writing – review and editing. **Masab Ali**: conceptualization, writing – original draft, writing – review and editing. **Taha Mehmood**: writing – original draft, writing – review and editing, project administration. **Muhammad Hassan**: project administration, writing – review and editing. **Aasim Ali**: writing – review and editing, visualization. **Amina Jamil**: visualization, writing – review and editing. **Muhammad Umar Shahid**: writing – review and editing, formal analysis. **Areej Asif**: writing – review and editing, methodology. **Aliha Wahab**: writing – review and editing, formal analysis. **Muhammad Husnain Ahmad**: validation, writing – original draft. **Noor Anwar**: data curation, project administration. **Hassaan Ahmed Bhatti**: investigation, validation.

## Funding

The authors have nothing to report.

## Ethics Statement

No ethical approval was required for this study design, as all data were obtained from publicly available, deidentified sources.

## Consent

The authors have nothing to report.

## Conflicts of Interest

The authors declare no conflicts of interest.

## Supporting information




**Table S1**: Annual number of deaths involving pneumonia and cerebrovascular disease, by sex and by race and ethnicity, adults aged 55 years and older, United States, 1999–2020.
**Table S2**: Annual number of deaths by place of death, adults aged 55 years and older, United States, 1999–2020.
**Table S3**: Annual percent change and average annual percent change in age‐adjusted mortality rates, overall and by stratum, adults aged 55 years and older, United States, 1999–2020.
**Table S4**: Age‐adjusted mortality rates per 100,000 populations, overall and by sex, adults aged 55 years and older, United States, 1999–2020.
**Table S5**: Deaths and age‐adjusted mortality rates per 100,000 populations by age group, adults aged 55 years and older, United States, 1999–2020.
**Table S6**: Age‐adjusted mortality rates per 100,000 populations by race and ethnicity, adults aged 55 years and older, United States, 1999–2020
**Table S7**: Deaths and pooled age‐adjusted mortality rates per 100,000 populations by state, adults aged 55 years and older, United States, 1999–2020.
**Table S8**: Age‐adjusted mortality rates per 100,000 populations by census region, adults aged 55 years and older, United States, 1999–2020.
**Table S9**: Age‐adjusted mortality rates per 100,000 populations by urbanization, adults aged 55 years and older, United States, 1999–2020.
**Table S10**: Annual deaths and age‐adjusted mortality rates under the primary (J18) and broader (J12–J18) pneumonia definitions, adults aged 55 years and older, United States, 1999–2020.
**Table S11**: Joinpoint estimates for the overall series under the primary (J18) and broader (J12–J18) pneumonia definitions, adults aged 55 years and older, United States, 1999–2020.
**Table S12**: Age‐adjusted mortality rates per 100,000 in 1999, 2019, and 2020, percent change, and average annual percent change (AAPC), overall and by stratum, adults aged 55 years and older, United States.
**Table S13**: Primary and sensitivity analyses of deaths involving pneumonia and cerebrovascular disease, adults aged 55 years and older, United States, 1999–2020.

## Data Availability

The data supporting the findings of this study are openly available in CDC WONDER at https://wonder.cdc.gov/.
