# Supplementary material for: Trends and Disparities in Mortality From Pneumonia and Cerebrovascular Disease in the United States (1999–2020)
Source: Brain Behav. 2026 Oct 22;16(10):e71849. doi: 10.1002/brb3.71849 (PMC13691420; doi:10.1002/brb3.71849)
Supplement: Supplementary file 1 [file BRB3-16-e71849-s001.docx]

**Supplemental Table 1. Annual Number of Deaths Involving Pneumonia and Cerebrovascular Disease, by Sex and by Race and Ethnicity, Adults Aged 55 Years and Older, United States, 1999 to 2020**

| **Year** | **Deaths, n** | **Women, n (%)** | **Men, n (%)** | **NH White, n (%)** | **NH Black, n (%)** | **NH Asian or Pacific Islander, n (%)** | **NH American Indian or Alaska Native, n (%)** | **Hispanic or Latino, n (%)** | **Population aged 55+** |
| --- | --- | --- | --- | --- | --- | --- | --- | --- | --- |
| 1999 | 26,309 | 14,150 (53.8) | 12,159 (46.2) | 21,872 (83.1) | 2,728 (10.4) | 592 (2.3) | 82 (0.3) | 978 (3.7) | 58,575,867 |
| 2000 | 24,994 | 13,061 (52.3) | 11,933 (47.7) | 20,543 (82.2) | 2,769 (11.1) | 547 (2.2) | 95 (0.4) | 974 (3.9) | 59,266,437 |
| 2001 | 23,393 | 12,521 (53.5) | 10,872 (46.5) | 19,072 (81.5) | 2,598 (11.1) | 598 (2.6) | 72 (0.3) | 988 (4.2) | 60,395,586 |
| 2002 | 22,696 | 12,074 (53.2) | 10,622 (46.8) | 18,505 (81.5) | 2,496 (11.0) | 583 (2.6) | 85 (0.4) | 982 (4.3) | 62,225,539 |
| 2003 | 21,005 | 11,102 (52.9) | 9,903 (47.1) | 17,104 (81.4) | 2,288 (10.9) | 608 (2.9) | 72 (0.3) | 901 (4.3) | 63,872,474 |
| 2004 | 19,125 | 10,045 (52.5) | 9,080 (47.5) | 15,452 (80.8) | 2,095 (11.0) | 596 (3.1) | 59 (0.3) | 896 (4.7) | 65,508,623 |
| 2005 | 17,866 | 9,368 (52.4) | 8,498 (47.6) | 14,331 (80.2) | 1,974 (11.0) | 548 (3.1) | 71 (0.4) | 912 (5.1) | 67,291,295 |
| 2006 | 15,477 | 8,051 (52.0) | 7,426 (48.0) | 12,199 (78.8) | 1,745 (11.3) | 540 (3.5) | 77 (0.5) | 884 (5.7) | 69,094,220 |
| 2007 | 14,310 | 7,296 (51.0) | 7,014 (49.0) | 11,285 (78.9) | 1,694 (11.8) | 482 (3.4) | 62 (0.4) | 776 (5.4) | 70,954,145 |
| 2008 | 12,786 | 6,858 (53.6) | 5,928 (46.4) | 9,995 (78.2) | 1,540 (12.0) | 480 (3.8) | 68 (0.5) | 683 (5.3) | 72,934,684 |
| 2009 | 10,994 | 5,695 (51.8) | 5,299 (48.2) | 8,435 (76.7) | 1,356 (12.3) | 447 (4.1) | 58 (0.5) | 678 (6.2) | 75,028,775 |
| 2010 | 10,737 | 5,503 (51.3) | 5,234 (48.7) | 8,208 (76.4) | 1,394 (13.0) | 398 (3.7) | 49 (0.5) | 669 (6.2) | 76,750,713 |
| 2011 | 10,813 | 5,623 (52.0) | 5,190 (48.0) | 8,251 (76.3) | 1,331 (12.3) | 438 (4.1) | 64 (0.6) | 707 (6.5) | 79,456,281 |
| 2012 | 10,058 | 5,207 (51.8) | 4,851 (48.2) | 7,587 (75.4) | 1,242 (12.3) | 416 (4.1) | 45 (0.4) | 739 (7.3) | 81,731,558 |
| 2013 | 9,788 | 4,993 (51.0) | 4,795 (49.0) | 7,279 (74.4) | 1,259 (12.9) | 445 (4.5) | 48 (0.5) | 730 (7.5) | 84,020,505 |
| 2014 | 9,131 | 4,539 (49.7) | 4,592 (50.3) | 6,756 (74.0) | 1,196 (13.1) | 425 (4.7) | 60 (0.7) | 669 (7.3) | 86,320,792 |
| 2015 | 9,171 | 4,490 (49.0) | 4,681 (51.0) | 6,876 (75.0) | 1,175 (12.8) | 385 (4.2) | 49 (0.5) | 657 (7.2) | 88,638,671 |
| 2016 | 8,654 | 4,244 (49.0) | 4,410 (51.0) | 6,292 (72.7) | 1,195 (13.8) | 403 (4.7) | 46 (0.5) | 690 (8.0) | 90,707,339 |
| 2017 | 8,611 | 4,130 (48.0) | 4,481 (52.0) | 6,228 (72.3) | 1,190 (13.8) | 404 (4.7) | 82 (1.0) | 682 (7.9) | 92,854,337 |
| 2018 | 8,330 | 3,978 (47.8) | 4,352 (52.2) | 6,001 (72.0) | 1,161 (13.9) | 420 (5.0) | 40 (0.5) | 688 (8.3) | 94,703,829 |
| 2019 | 8,132 | 3,800 (46.7) | 4,332 (53.3) | 5,865 (72.1) | 1,151 (14.2) | 399 (4.9) | 45 (0.6) | 653 (8.0) | 96,506,800 |
| 2020 | 14,380 | 6,290 (43.7) | 8,090 (56.3) | 9,142 (63.6) | 2,562 (17.8) | 795 (5.5) | 120 (0.8) | 1,725 (12.0) | 98,063,042 |

NH = non-Hispanic. Race and Hispanic origin were extracted as separate CDC WONDER queries and merged into mutually exclusive categories. The five race and ethnicity columns sum to 316,076 rather than 316,760 because 684 deaths (0.2%) had Hispanic origin not stated and were excluded from that stratification only. Percentages are of all deaths in that year. Population denominators are the United States resident population aged 55 years and older.

**Supplemental Table 2. Annual Number of Deaths by Place of Death, Adults Aged 55 Years and Older, United States, 1999 to 2020**

| **Year** | **Medical facility, n (%)** | **Nursing home or long term care, n (%)** | **Hospice facility, n (%)** | **Decedent's home, n (%)** | **Other or unspecified, n (%)** | **Total deaths, n** |
| --- | --- | --- | --- | --- | --- | --- |
| 1999 | 14,399 (54.7) | 10,362 (39.4) | Not applicable | 1,228 (4.7) | 320 (1.2) | 26,309 |
| 2000 | 13,752 (55.0) | 9,622 (38.5) | Not applicable | 1,277 (5.1) | 343 (1.4) | 24,994 |
| 2001 | 12,841 (54.9) | 8,956 (38.3) | Not applicable | 1,228 (5.2) | 368 (1.6) | 23,393 |
| 2002 | 12,621 (55.6) | 8,375 (36.9) | Not applicable | 1,297 (5.7) | 403 (1.8) | 22,696 |
| 2003 | 11,374 (54.1) | 7,875 (37.5) | 20 (0.1) | 1,256 (6.0) | 480 (2.3) | 21,005 |
| 2004 | 10,431 (54.5) | 7,001 (36.6) | 59 (0.3) | 1,176 (6.1) | 458 (2.4) | 19,125 |
| 2005 | 9,855 (55.2) | 6,348 (35.5) | 146 (0.8) | 1,092 (6.1) | 425 (2.4) | 17,866 |
| 2006 | 8,682 (56.1) | 5,198 (33.6) | 224 (1.4) | 1,042 (6.7) | 331 (2.1) | 15,477 |
| 2007 | 8,066 (56.4) | 4,636 (32.4) | 258 (1.8) | 1,005 (7.0) | 345 (2.4) | 14,310 |
| 2008 | 7,152 (55.9) | 4,028 (31.5) | 297 (2.3) | 875 (6.8) | 434 (3.4) | 12,786 |
| 2009 | 6,035 (54.9) | 3,348 (30.5) | 296 (2.7) | 828 (7.5) | 487 (4.4) | 10,994 |
| 2010 | 5,919 (55.1) | 3,335 (31.1) | 405 (3.8) | 819 (7.6) | 259 (2.4) | 10,737 |
| 2011 | 5,923 (54.8) | 3,367 (31.1) | 432 (4.0) | 806 (7.5) | 285 (2.6) | 10,813 |
| 2012 | 5,511 (54.8) | 2,987 (29.7) | 529 (5.3) | 759 (7.5) | 272 (2.7) | 10,058 |
| 2013 | 5,295 (54.1) | 2,898 (29.6) | 526 (5.4) | 772 (7.9) | 297 (3.0) | 9,788 |
| 2014 | 5,007 (54.8) | 2,547 (27.9) | 617 (6.8) | 782 (8.6) | 178 (1.9) | 9,131 |
| 2015 | 4,960 (54.1) | 2,487 (27.1) | 756 (8.2) | 781 (8.5) | 187 (2.0) | 9,171 |
| 2016 | 4,784 (55.3) | 2,182 (25.2) | 726 (8.4) | 800 (9.2) | 162 (1.9) | 8,654 |
| 2017 | 4,763 (55.3) | 2,114 (24.5) | 749 (8.7) | 772 (9.0) | 213 (2.5) | 8,611 |
| 2018 | 4,711 (56.6) | 1,929 (23.2) | 772 (9.3) | 751 (9.0) | 167 (2.0) | 8,330 |
| 2019 | 4,560 (56.1) | 1,815 (22.3) | 782 (9.6) | 790 (9.7) | 185 (2.3) | 8,132 |
| 2020 | 8,956 (62.3) | 2,775 (19.3) | 1,126 (7.8) | 1,213 (8.4) | 310 (2.2) | 14,380 |
| **Total** | **175,597 (55.4)** | **104,185 (32.9)** | **8,720 (2.8)** | **21,349 (6.7)** | **6,909 (2.2)** | **316,760** |

Percentages are of all deaths in that year. Hospice facility was not available as a separate place of death category in CDC WONDER before 2003; deaths occurring in hospice in 1999 to 2002 were not separately identifiable and are distributed among the remaining categories. The other or unspecified column is derived as the annual total minus the four named categories.

**Supplemental Table 3. Annual Percent Change and Average Annual Percent Change in Age Adjusted Mortality Rates, Overall and by Stratum, Adults Aged 55 Years and Older, United States, 1999 to 2020**

| **Stratum** | **Group** | **Year interval** | **Measure** | **Estimate, % (95% CI)** | **p value** |
| --- | --- | --- | --- | --- | --- |
| Overall | Overall | 1999 to 2003 | APC | -6.48 (-9.93 to 4.62) | 0.109 |
| Overall | Overall | 2003 to 2009 | APC | -11.61 (-17.30 to -4.47) | 0.002 |
| Overall | Overall | 2009 to 2018 | APC | -6.41 (-14.65 to -1.50) | 0.018 |
| Overall | Overall | 2018 to 2020 | APC | 28.85 (14.78 to 39.73) | <0.001 |
| Overall | Overall | 1999 to 2020 | AAPC | -5.09 (-5.92 to -4.26) | <0.001 |
| Sex | Men | 1999 to 2004 | APC | -7.46 (-10.17 to 0.90) | 0.062 |
| Sex | Men | 2004 to 2009 | APC | -12.80 (-18.34 to -4.80) | 0.004 |
| Sex | Men | 2009 to 2018 | APC | -6.21 (-13.93 to -1.40) | 0.025 |
| Sex | Men | 2018 to 2020 | APC | 32.62 (18.06 to 43.43) | <0.001 |
| Sex | Men | 1999 to 2020 | AAPC | -5.03 (-5.83 to -4.37) | <0.001 |
| Sex | Women | 1999 to 2018 | APC | -8.59 (-9.74 to -8.03) | 0.003 |
| Sex | Women | 2018 to 2020 | APC | 23.62 (-3.65 to 35.83) | 0.088 |
| Sex | Women | 1999 to 2020 | AAPC | -5.93 (-7.70 to -5.23) | <0.001 |
| Age | 55 to 74 years | 1999 to 2012 | APC | -8.47 (-10.90 to -5.37) | 0.013 |
| Age | 55 to 74 years | 2012 to 2018 | APC | -2.24 (-7.99 to 6.27) | 0.416 |
| Age | 55 to 74 years | 2018 to 2020 | APC | 40.45 (23.76 to 55.46) | <0.001 |
| Age | 55 to 74 years | 1999 to 2020 | AAPC | -2.85 (-3.70 to -2.34) | <0.001 |
| Age | 75 years and older | 1999 to 2003 | APC | -6.62 (-9.54 to 1.09) | 0.064 |
| Age | 75 years and older | 2003 to 2009 | APC | -11.86 (-16.84 to -9.61) | <0.001 |
| Age | 75 years and older | 2009 to 2018 | APC | -7.12 (-12.18 to -2.48) | 0.008 |
| Age | 75 years and older | 2018 to 2020 | APC | 21.59 (6.06 to 30.21) | 0.006 |
| Age | 75 years and older | 1999 to 2020 | AAPC | -6.03 (-6.98 to -5.42) | <0.001 |
| Race and ethnicity | NH American Indian or Alaska Native | 1999 to 2018 | APC | -7.41 (-11.33 to -5.84) | 0.010 |
| Race and ethnicity | NH American Indian or Alaska Native | 2018 to 2020 | APC | 37.00 (-2.42 to 66.16) | 0.080 |
| Race and ethnicity | NH American Indian or Alaska Native | 1999 to 2020 | AAPC | -3.89 (-6.44 to -2.27) | <0.001 |
| Race and ethnicity | NH Asian or Pacific Islander | 1999 to 2018 | APC | -9.07 (-9.88 to -8.42) | <0.001 |
| Race and ethnicity | NH Asian or Pacific Islander | 2018 to 2020 | APC | 33.38 (16.90 to 43.42) | <0.001 |
| Race and ethnicity | NH Asian or Pacific Islander | 1999 to 2020 | AAPC | -5.69 (-6.57 to -5.11) | <0.001 |
| Race and ethnicity | NH Black or African American | 1999 to 2018 | APC | -8.23 (-9.17 to -7.56) | <0.001 |
| Race and ethnicity | NH Black or African American | 2018 to 2020 | APC | 47.62 (26.95 to 60.70) | <0.001 |
| Race and ethnicity | NH Black or African American | 1999 to 2020 | AAPC | -3.98 (-5.08 to -3.38) | <0.001 |
| Race and ethnicity | NH White | 1999 to 2004 | APC | -7.41 (-9.40 to -1.24) | 0.039 |
| Race and ethnicity | NH White | 2004 to 2009 | APC | -12.62 (-17.51 to -9.63) | <0.001 |
| Race and ethnicity | NH White | 2009 to 2018 | APC | -6.22 (-8.65 to -3.01) | 0.004 |
| Race and ethnicity | NH White | 2018 to 2020 | APC | 21.77 (10.55 to 28.85) | <0.001 |
| Race and ethnicity | NH White | 1999 to 2020 | AAPC | -5.75 (-6.42 to -5.20) | <0.001 |
| Race and ethnicity | Hispanic or Latino | 1999 to 2018 | APC | -8.32 (-9.71 to -7.29) | <0.001 |
| Race and ethnicity | Hispanic or Latino | 2018 to 2020 | APC | 51.94 (21.74 to 68.43) | <0.001 |
| Race and ethnicity | Hispanic or Latino | 1999 to 2020 | AAPC | -3.80 (-5.21 to -2.95) | <0.001 |
| Urbanization | Metropolitan | 1999 to 2011 | APC | -9.67 (-14.52 to -6.32) | 0.003 |
| Urbanization | Metropolitan | 2011 to 2018 | APC | -6.22 (-10.44 to -1.04) | 0.025 |
| Urbanization | Metropolitan | 2018 to 2020 | APC | 29.91 (15.68 to 40.60) | <0.001 |
| Urbanization | Metropolitan | 1999 to 2020 | AAPC | -5.31 (-6.14 to -4.76) | <0.001 |
| Urbanization | Non-metropolitan | 1999 to 2018 | APC | -8.37 (-9.11 to -7.88) | <0.001 |
| Urbanization | Non-metropolitan | 2018 to 2020 | APC | 27.97 (9.61 to 37.59) | 0.004 |
| Urbanization | Non-metropolitan | 1999 to 2020 | AAPC | -5.41 (-6.46 to -4.91) | <0.001 |
| Census region | Northeast | 1999 to 2009 | APC | -9.84 (-13.94 to -8.74) | <0.001 |
| Census region | Northeast | 2009 to 2018 | APC | -5.65 (-8.11 to -0.70) | 0.034 |
| Census region | Northeast | 2018 to 2020 | APC | 27.47 (10.67 to 37.92) | <0.001 |
| Census region | Northeast | 1999 to 2020 | AAPC | -4.99 (-5.98 to -4.47) | <0.001 |
| Census region | Midwest | 1999 to 2018 | APC | -8.52 (-9.34 to -7.99) | <0.001 |
| Census region | Midwest | 2018 to 2020 | APC | 33.25 (10.63 to 45.17) | 0.003 |
| Census region | Midwest | 1999 to 2020 | AAPC | -5.18 (-6.37 to -4.58) | <0.001 |
| Census region | South | 1999 to 2010 | APC | -9.53 (-13.96 to -7.58) | 0.001 |
| Census region | South | 2010 to 2018 | APC | -6.21 (-9.58 to -1.00) | 0.027 |
| Census region | South | 2018 to 2020 | APC | 30.08 (14.99 to 41.27) | <0.001 |
| Census region | South | 1999 to 2020 | AAPC | -5.05 (-5.95 to -4.52) | <0.001 |
| Census region | West | 1999 to 2004 | APC | -6.75 (-8.77 to -2.34) | 0.019 |
| Census region | West | 2004 to 2009 | APC | -14.38 (-19.21 to -11.82) | <0.001 |
| Census region | West | 2009 to 2018 | APC | -7.36 (-9.56 to -3.81) | 0.005 |
| Census region | West | 2018 to 2020 | APC | 20.82 (5.23 to 29.28) | 0.007 |
| Census region | West | 1999 to 2020 | AAPC | -6.61 (-7.56 to -6.05) | <0.001 |

APC = annual percent change; AAPC = average annual percent change; CI = confidence interval; NH = non-Hispanic. Estimates were derived by Joinpoint regression of the annual age adjusted mortality rate series. Year intervals differ between rows because joinpoints were estimated independently within each stratum; each interval corresponds to a segment bounded by the joinpoints identified for that stratum. An estimate is statistically significant when p < 0.05. P values below 0.001 are reported as <0.001.

**Supplemental Table 4. Age Adjusted Mortality Rates per 100,000 Population, Overall and by Sex, Adults Aged 55 Years and Older, United States, 1999 to 2020**

| **Year** | **Men** | **Women** | **Overall** |
| --- | --- | --- | --- |
| 1999 | 58.1 | 37.8 | 45.4 |
| 2000 | 55.9 | 34.4 | 42.5 |
| 2001 | 49.7 | 32.7 | 39.2 |
| 2002 | 47.9 | 31.2 | 37.5 |
| 2003 | 43.5 | 28.3 | 34.1 |
| 2004 | 38.9 | 25.4 | 30.6 |
| 2005 | 35.4 | 23.3 | 28.0 |
| 2006 | 30.0 | 19.8 | 23.8 |
| 2007 | 27.6 | 17.5 | 21.5 |
| 2008 | 22.6 | 16.2 | 18.8 |
| 2009 | 19.8 | 13.2 | 15.9 |
| 2010 | 19.0 | 12.6 | 15.2 |
| 2011 | 18.1 | 12.6 | 14.8 |
| 2012 | 16.4 | 11.4 | 13.5 |
| 2013 | 15.6 | 10.7 | 12.8 |
| 2014 | 14.4 | 9.6 | 11.6 |
| 2015 | 14.3 | 9.3 | 11.4 |
| 2016 | 13.0 | 8.7 | 10.5 |
| 2017 | 12.8 | 8.2 | 10.2 |
| 2018 | 11.9 | 7.8 | 9.6 |
| 2019 | 11.6 | 7.3 | 9.1 |
| 2020 | 20.8 | 12.0 | 15.8 |

Rates are age adjusted to the 2000 United States standard population and expressed per 100,000. No pooled multi year row is shown, because the unweighted mean of annual rates is not itself an age adjusted rate for the period.

**Supplemental Table 5. Deaths and Age Adjusted Mortality Rates per 100,000 Population by Age Group, Adults Aged 55 Years and Older, United States, 1999 to 2020**

| **Year** | **Deaths, 55 to 74 years** | **Deaths, 75 years and older** | **AAMR, 55 to 74 years** | **AAMR, 75 years and older** |
| --- | --- | --- | --- | --- |
| 1999 | 4,808 | 21,501 | 11.3 | 132.0 |
| 2000 | 4,548 | 20,446 | 10.6 | 123.4 |
| 2001 | 4,384 | 19,009 | 10.2 | 112.7 |
| 2002 | 4,134 | 18,562 | 9.5 | 108.6 |
| 2003 | 3,899 | 17,106 | 8.8 | 98.5 |
| 2004 | 3,658 | 15,467 | 8.1 | 87.9 |
| 2005 | 3,370 | 14,496 | 7.3 | 80.7 |
| 2006 | 3,125 | 12,352 | 6.6 | 67.5 |
| 2007 | 2,967 | 11,343 | 6.1 | 60.7 |
| 2008 | 2,662 | 10,124 | 5.3 | 53.4 |
| 2009 | 2,287 | 8,707 | 4.3 | 45.1 |
| 2010 | 2,373 | 8,364 | 4.4 | 42.7 |
| 2011 | 2,405 | 8,408 | 4.3 | 41.7 |
| 2012 | 2,317 | 7,741 | 3.9 | 37.7 |
| 2013 | 2,343 | 7,445 | 3.8 | 35.6 |
| 2014 | 2,381 | 6,750 | 3.7 | 31.6 |
| 2015 | 2,343 | 6,828 | 3.6 | 31.4 |
| 2016 | 2,503 | 6,151 | 3.7 | 27.9 |
| 2017 | 2,497 | 6,114 | 3.6 | 27.1 |
| 2018 | 2,569 | 5,761 | 3.6 | 24.8 |
| 2019 | 2,589 | 5,543 | 3.5 | 23.5 |
| 2020 | 5,184 | 9,196 | 6.9 | 38.4 |
| **Total deaths** | **69,346** | **247,414** |  |  |

AAMR = age adjusted mortality rate, standardized to the 2000 United States standard population and expressed per 100,000. The two age groups sum to the full cohort of 316,760 deaths.

**Supplemental Table 6. Age Adjusted Mortality Rates per 100,000 Population by Race and Ethnicity, Adults Aged 55 Years and Older, United States, 1999 to 2020**

| **Year** | **NH White** | **NH Black or African American** | **NH American Indian or Alaska Native** | **Hispanic or Latino** | **NH Asian or Pacific Islander** |
| --- | --- | --- | --- | --- | --- |
| 1999 | 43.8 | 60.0 | 43.2 | 40.7 | 55.0 |
| 2000 | 40.7 | 60.2 | 44.9 | 39.3 | 47.8 |
| 2001 | 37.4 | 55.6 | 33.6 | 37.2 | 46.9 |
| 2002 | 35.9 | 53.0 | 37.8 | 35.2 | 43.5 |
| 2003 | 32.7 | 47.9 | 32.3 | 30.5 | 42.4 |
| 2004 | 29.2 | 42.7 | 23.9 | 28.7 | 39.3 |
| 2005 | 26.7 | 39.2 | 31.4 | 27.5 | 33.0 |
| 2006 | 22.4 | 34.0 | 31.1 | 25.5 | 31.1 |
| 2007 | 20.3 | 32.0 | 23.7 | 21.1 | 25.7 |
| 2008 | 17.7 | 28.0 | 23.3 | 17.4 | 24.1 |
| 2009 | 14.7 | 24.1 | 19.7 | 16.3 | 21.4 |
| 2010 | 14.0 | 24.3 | 16.4 | 15.6 | 17.8 |
| 2011 | 13.8 | 22.2 | 19.7 | 15.0 | 18.2 |
| 2012 | 12.5 | 19.9 | 12.6 | 14.7 | 16.0 |
| 2013 | 11.8 | 19.2 | 12.8 | 13.6 | 15.9 |
| 2014 | 10.8 | 17.5 | 15.7 | 11.7 | 13.9 |
| 2015 | 10.8 | 16.4 | 11.0 | 10.6 | 11.6 |
| 2016 | 9.7 | 16.2 | 11.2 | 10.7 | 11.6 |
| 2017 | 9.4 | 15.3 | 17.4 | 9.9 | 10.8 |
| 2018 | 8.9 | 14.4 | 8.5 | 9.6 | 10.5 |
| 2019 | 8.6 | 13.7 | 8.2 | 8.6 | 9.6 |
| 2020 | 13.2 | 29.5 | 21.3 | 21.0 | 17.7 |

NH = non-Hispanic. Rates are age adjusted to the 2000 United States standard population and expressed per 100,000. No pooled multi year row is shown, because the unweighted mean of annual rates is not itself an age adjusted rate for the period.

**Supplemental Table 7. Deaths and Pooled Age Adjusted Mortality Rates per 100,000 Population by State, Adults Aged 55 Years and Older, United States, 1999 to 2020**

| **Rank** | **State** | **Deaths, n** | **% of all deaths** | **Pooled AAMR, 1999 to 2020** |
| --- | --- | --- | --- | --- |
| 1 | West Virginia | 3,143 | 1.0 | 28.4 |
| 2 | California | 47,374 | 15.0 | 28.1 |
| 3 | Oklahoma | 5,171 | 1.6 | 27.1 |
| 4 | Kentucky | 5,787 | 1.8 | 27.0 |
| 5 | Tennessee | 8,223 | 2.6 | 26.4 |
| 6 | Mississippi | 3,716 | 1.2 | 26.0 |
| 7 | North Carolina | 11,681 | 3.7 | 25.6 |
| 8 | Maryland | 6,975 | 2.2 | 25.3 |
| 9 | District of Columbia | 714 | 0.2 | 25.3 |
| 10 | Alabama | 5,876 | 1.9 | 24.2 |
| 11 | Arkansas | 3,720 | 1.2 | 23.7 |
| 12 | Ohio | 15,114 | 4.8 | 23.7 |
| 13 | Vermont | 831 | 0.3 | 23.5 |
| 14 | South Carolina | 5,285 | 1.7 | 23.2 |
| 15 | Rhode Island | 1,465 | 0.5 | 22.4 |
| 16 | North Dakota | 954 | 0.3 | 22.3 |
| 17 | Texas | 21,628 | 6.8 | 22.2 |
| 18 | Hawaii | 1,774 | 0.6 | 22.1 |
| 19 | Washington | 7,079 | 2.2 | 21.9 |
| 20 | South Dakota | 1,071 | 0.3 | 21.7 |
| 21 | Nebraska | 2,169 | 0.7 | 21.1 |
| 22 | Indiana | 6,716 | 2.1 | 20.6 |
| 23 | Missouri | 6,593 | 2.1 | 20.2 |
| 24 | Minnesota | 5,726 | 1.8 | 20.1 |
| 25 | Illinois | 12,830 | 4.1 | 19.9 |
| 26 | Delaware | 955 | 0.3 | 19.9 |
| 27 | Massachusetts | 7,438 | 2.3 | 19.7 |
| 28 | Virginia | 6,972 | 2.2 | 19.1 |
| 29 | Louisiana | 4,049 | 1.3 | 19.0 |
| 30 | Alaska | 342 | 0.1 | 18.9 |
| 31 | Michigan | 9,866 | 3.1 | 18.6 |
| 32 | Idaho | 1,354 | 0.4 | 18.6 |
| 33 | Kansas | 2,916 | 0.9 | 18.6 |
| 34 | Connecticut | 3,904 | 1.2 | 18.4 |
| 35 | Iowa | 3,507 | 1.1 | 18.1 |
| 36 | Oregon | 3,809 | 1.2 | 18.1 |
| 37 | Georgia | 6,857 | 2.2 | 18.0 |
| 38 | Montana | 1,002 | 0.3 | 17.7 |
| 39 | New Mexico | 1,755 | 0.6 | 17.6 |
| 40 | Pennsylvania | 14,050 | 4.4 | 17.5 |
| 41 | Wyoming | 453 | 0.1 | 17.4 |
| 42 | Maine | 1,409 | 0.4 | 17.2 |
| 43 | Utah | 1,592 | 0.5 | 17.1 |
| 44 | New Jersey | 8,131 | 2.6 | 17.0 |
| 45 | Wisconsin | 5,282 | 1.7 | 16.7 |
| 46 | New Hampshire | 1,144 | 0.4 | 16.3 |
| 47 | New York | 16,932 | 5.3 | 15.9 |
| 48 | Colorado | 2,935 | 0.9 | 14.1 |
| 49 | Nevada | 1,361 | 0.4 | 11.9 |
| 50 | Florida | 13,980 | 4.4 | 10.9 |
| 51 | Arizona | 3,150 | 1.0 | 9.6 |

AAMR = age adjusted mortality rate, standardized to the 2000 United States standard population and expressed per 100,000. Rates are pooled across the whole 1999 to 2020 period and are therefore weighted towards the earlier years of the series, when rates were highest; they should not be compared directly with single year rates reported elsewhere. States are ordered from the highest to the lowest pooled rate.

**Supplemental Table 8. Age Adjusted Mortality Rates per 100,000 Population by Census Region, Adults Aged 55 Years and Older, United States, 1999 to 2020**

| **Year** | **Northeast** | **Midwest** | **South** | **West** |
| --- | --- | --- | --- | --- |
| 1999 | 37.2 | 44.3 | 45.3 | 55.7 |
| 2000 | 35.7 | 40.0 | 43.1 | 51.9 |
| 2001 | 32.9 | 37.1 | 39.7 | 47.5 |
| 2002 | 30.5 | 36.2 | 38.4 | 45.0 |
| 2003 | 27.2 | 33.7 | 34.4 | 41.6 |
| 2004 | 25.5 | 29.7 | 29.8 | 38.4 |
| 2005 | 23.0 | 28.6 | 27.7 | 33.3 |
| 2006 | 18.8 | 23.0 | 24.0 | 29.4 |
| 2007 | 17.3 | 21.3 | 21.7 | 25.6 |
| 2008 | 15.2 | 19.5 | 19.0 | 21.3 |
| 2009 | 12.7 | 16.0 | 16.5 | 17.5 |
| 2010 | 12.4 | 14.8 | 16.3 | 16.3 |
| 2011 | 12.9 | 15.4 | 14.9 | 15.9 |
| 2012 | 12.0 | 13.5 | 14.0 | 13.7 |
| 2013 | 11.3 | 12.7 | 13.4 | 13.1 |
| 2014 | 10.3 | 11.5 | 12.5 | 11.3 |
| 2015 | 10.6 | 11.1 | 12.1 | 11.2 |
| 2016 | 9.3 | 10.4 | 11.1 | 10.7 |
| 2017 | 9.1 | 10.2 | 10.4 | 10.6 |
| 2018 | 9.0 | 8.9 | 10.5 | 9.3 |
| 2019 | 8.6 | 9.4 | 9.4 | 8.9 |
| 2020 | 14.8 | 15.7 | 17.1 | 14.4 |

Rates are age adjusted to the 2000 United States standard population and expressed per 100,000. No pooled multi year row is shown, because the unweighted mean of annual rates is not itself an age adjusted rate for the period.

**Supplemental Table 9. Age Adjusted Mortality Rates per 100,000 Population by Urbanization, Adults Aged 55 Years and Older, United States, 1999 to 2020**

| **Year** | **Metropolitan** | **Non-metropolitan** | **Absolute difference** |
| --- | --- | --- | --- |
| 1999 | 44.2 | 50.3 | 6.1 |
| 2000 | 41.3 | 47.5 | 6.2 |
| 2001 | 38.1 | 43.7 | 5.6 |
| 2002 | 35.9 | 44.0 | 8.1 |
| 2003 | 33.0 | 38.9 | 5.9 |
| 2004 | 29.6 | 35.4 | 5.8 |
| 2005 | 26.8 | 33.6 | 6.8 |
| 2006 | 22.7 | 28.8 | 6.1 |
| 2007 | 20.6 | 25.4 | 4.8 |
| 2008 | 17.9 | 23.0 | 5.1 |
| 2009 | 15.1 | 19.4 | 4.3 |
| 2010 | 14.4 | 18.9 | 4.5 |
| 2011 | 14.2 | 17.7 | 3.5 |
| 2012 | 12.8 | 16.4 | 3.6 |
| 2013 | 12.1 | 15.8 | 3.7 |
| 2014 | 11.1 | 14.0 | 2.9 |
| 2015 | 10.9 | 13.8 | 2.9 |
| 2016 | 10.2 | 12.3 | 2.1 |
| 2017 | 9.9 | 11.5 | 1.6 |
| 2018 | 9.3 | 11.2 | 1.9 |
| 2019 | 8.9 | 10.6 | 1.7 |
| 2020 | 15.5 | 17.1 | 1.6 |

Rates are age adjusted to the 2000 United States standard population and expressed per 100,000. Metropolitan combines the large central, large fringe, medium and small metropolitan categories of the NCHS Urban Rural Classification Scheme for Counties; non-metropolitan combines the micropolitan and non-core categories. The absolute difference is the non-metropolitan rate minus the metropolitan rate.

**Supplemental Table 10. Annual Deaths and Age Adjusted Mortality Rates Under the Primary (J18) and Broader (J12 to J18) Pneumonia Definitions, Adults Aged 55 Years and Older, United States, 1999 to 2020**

| **Year** | **Deaths, primary (J18)** | **Deaths, broader (J12 to J18)** | **Deaths added by broader definition, n** | **Added deaths, % of broader total** | **AAMR, primary** | **AAMR, broader** |
| --- | --- | --- | --- | --- | --- | --- |
| 1999 | 26,309 | 27,190 | 881 | 3.2 | 45.4 | 46.90 |
| 2000 | 24,994 | 25,955 | 961 | 3.7 | 42.5 | 44.16 |
| 2001 | 23,393 | 24,234 | 841 | 3.5 | 39.2 | 40.55 |
| 2002 | 22,696 | 23,515 | 819 | 3.5 | 37.5 | 38.83 |
| 2003 | 21,005 | 21,834 | 829 | 3.8 | 34.1 | 35.45 |
| 2004 | 19,125 | 19,853 | 728 | 3.7 | 30.6 | 31.79 |
| 2005 | 17,866 | 18,527 | 661 | 3.6 | 28.0 | 29.08 |
| 2006 | 15,477 | 16,107 | 630 | 3.9 | 23.8 | 24.78 |
| 2007 | 14,310 | 14,888 | 578 | 3.9 | 21.5 | 22.35 |
| 2008 | 12,786 | 13,344 | 558 | 4.2 | 18.8 | 19.61 |
| 2009 | 10,994 | 11,589 | 595 | 5.1 | 15.9 | 16.70 |
| 2010 | 10,737 | 11,222 | 485 | 4.3 | 15.2 | 15.85 |
| 2011 | 10,813 | 11,362 | 549 | 4.8 | 14.8 | 15.61 |
| 2012 | 10,058 | 10,555 | 497 | 4.7 | 13.5 | 14.11 |
| 2013 | 9,788 | 10,334 | 546 | 5.3 | 12.8 | 13.50 |
| 2014 | 9,131 | 9,676 | 545 | 5.6 | 11.6 | 12.30 |
| 2015 | 9,171 | 9,804 | 633 | 6.5 | 11.4 | 12.15 |
| 2016 | 8,654 | 9,285 | 631 | 6.8 | 10.5 | 11.23 |
| 2017 | 8,611 | 9,288 | 677 | 7.3 | 10.2 | 10.96 |
| 2018 | 8,330 | 9,004 | 674 | 7.5 | 9.6 | 10.31 |
| 2019 | 8,132 | 8,854 | 722 | 8.2 | 9.1 | 9.96 |
| 2020 | 14,380 | 15,861 | 1,481 | 9.3 | 15.8 | 17.43 |
| **Total** | **316,760** | **332,281** | **15,521** | **4.7** |  |  |

AAMR = age adjusted mortality rate per 100,000, standardized to the 2000 United States standard population. Both definitions were combined with cerebrovascular disease (I60 to I69) in the multiple cause of death field. The primary definition is a subset of the broader definition, so deaths added by the broader definition are those with a J12 to J17 code and no J18 code. The added deaths equal 4.9% of the primary total (15,521 of 316,760). Rates for the primary definition are available to one decimal place and for the broader definition to two decimal places.

**Supplemental Table 11. Joinpoint Estimates for the Overall Series Under the Primary (J18) and Broader (J12 to J18) Pneumonia Definitions, Adults Aged 55 Years and Older, United States, 1999 to 2020**

| **Definition** | **Year interval** | **Measure** | **Estimate, % (95% CI)** | **p value** |
| --- | --- | --- | --- | --- |
| Primary (J18) | 1999 to 2003 | APC | −6.48 (−9.93 to 4.62) | 0.109 |
| Primary (J18) | 2003 to 2009 | APC | −11.61 (−17.30 to −4.47) | 0.002 |
| Primary (J18) | 2009 to 2018 | APC | −6.41 (−14.65 to −1.50) | 0.018 |
| Primary (J18) | 2018 to 2020 | APC | 28.85 (14.78 to 39.73) | <0.001 |
| Primary (J18) | 1999 to 2020 | AAPC | −5.09 (−5.92 to −4.26) | <0.001 |
| Broader (J12 to J18) | 1999 to 2003 | APC | −6.34 (−9.62 to 2.27) | 0.086 |
| Broader (J12 to J18) | 2003 to 2009 | APC | −11.58 (−17.06 to −4.60) | 0.004 |
| Broader (J12 to J18) | 2009 to 2018 | APC | −6.07 (−13.61 to −0.83) | 0.030 |
| Broader (J12 to J18) | 2018 to 2020 | APC | 29.08 (12.58 to 39.01) | <0.001 |
| Broader (J12 to J18) | 1999 to 2020 | AAPC | −4.89 (−5.86 to −4.21) | <0.001 |

APC = annual percent change; AAPC = average annual percent change; CI = confidence interval. Joinpoint settings were identical for the two analyses (maximum of four joinpoints, permutation test). Both analyses identified three joinpoints (2003, 2009 and 2018). An estimate is statistically significant when p is less than 0.05.

**Supplemental Table 12. Age adjusted mortality rates per 100,000 in 1999, 2019 and 2020, percent change, and average annual percent change (AAPC), overall and by stratum, adults aged 55 years and older, United States**

| **Stratum** | **AAMR 1999** | **AAMR 2019** | **AAMR 2020** | **% change 1999 to 2019** | **% change 2019 to 2020** | **AAPC, % (95% CI)** |
| --- | --- | --- | --- | --- | --- | --- |
| **Overall** | | | | | | |
| Overall | 45.4 | 9.1 | 15.8 | −80.0 | +73.6 | −5.09 (−5.92 to −4.26) |
| **Sex** | | | | | | |
| Men | 58.1 | 11.6 | 20.8 | −80.0 | +79.3 | −5.03 (−5.83 to −4.37) |
| Women | 37.8 | 7.3 | 12.0 | −80.7 | +64.4 | −5.93 (−7.70 to −5.23) |
| **Age group** | | | | | | |
| 55 to 74 years | 11.3 | 3.5 | 6.9 | −69.0 | +97.1 | −2.85 (−3.70 to −2.34) |
| 75 years and older | 132.0 | 23.5 | 38.4 | −82.2 | +63.4 | −6.03 (−6.98 to −5.42) |
| **Race and ethnicity** | | | | | | |
| NH White | 43.8 | 8.6 | 13.2 | −80.4 | +53.5 | −5.75 (−6.42 to −5.20) |
| NH Black or African American | 60.0 | 13.7 | 29.5 | −77.2 | +115.3 | −3.98 (−5.08 to −3.38) |
| NH American Indian or Alaska Native | 43.2 | 8.2 | 21.3 | −81.0 | +159.8 | −3.89 (−6.44 to −2.27) |
| NH Asian or Pacific Islander | 55.0 | 9.6 | 17.7 | −82.5 | +84.4 | −5.69 (−6.57 to −5.11) |
| Hispanic or Latino | 40.7 | 8.6 | 21.0 | −78.9 | +144.2 | −3.80 (−5.21 to −2.95) |
| **Urbanization** | | | | | | |
| Metropolitan | 44.2 | 8.9 | 15.5 | −79.9 | +74.2 | −5.31 (−6.14 to −4.76) |
| Non metropolitan | 50.3 | 10.6 | 17.1 | −78.9 | +61.3 | −5.41 (−6.46 to −4.91) |
| **Census region** | | | | | | |
| Northeast | 37.2 | 8.6 | 14.8 | −76.9 | +72.1 | −4.99 (−5.98 to −4.47) |
| Midwest | 44.3 | 9.4 | 15.7 | −78.8 | +67.0 | −5.18 (−6.37 to −4.58) |
| South | 45.3 | 9.4 | 17.1 | −79.2 | +81.9 | −5.05 (−5.95 to −4.52) |
| West | 55.7 | 8.9 | 14.4 | −84.0 | +61.8 | −6.61 (−7.56 to −6.05) |

AAMR = age adjusted mortality rate per 100,000 (2000 United States standard population); AAPC = average annual percent change, 1999 to 2020 (all p less than 0.001); NH = non-Hispanic. Percent changes were calculated from the rates shown. Race and ethnicity groups exclude 684 deaths with Hispanic origin not stated. Rates for American Indian or Alaska Native decedents rest on small numbers and are unstable. Segment level APCs are in Supplemental Table 3.

**Supplemental Table 13. Primary and sensitivity analyses of deaths involving pneumonia and cerebrovascular disease, adults aged 55 years and older, United States, 1999 to 2020**

| **Measure** | **Primary definition (J18)** | **Broader definition (J12 to J18)** |
| --- | --- | --- |
| Deaths, n | 316,760 | 332,281 |
| Deaths added by broader definition, n (%) | Not applicable | 15,521 (4.9% more than primary) |
| AAMR 1999 (per 100,000) | 45.4 | 46.90 |
| AAMR 2019 | 9.1 | 9.96 |
| AAMR 2020 | 15.8 | 17.43 |
| Change 1999 to 2019, % | −80.0 | −78.8 |
| Change 2019 to 2020, % | +73.6 | +75.0 |
| Joinpoints | 2003, 2009, 2018 | 2003, 2009, 2018 |
| APC 2018 to 2020, % (95% CI) | 28.85 (14.78 to 39.73) | 29.08 (12.58 to 39.01) |
| AAPC 1999 to 2020, % (95% CI) | −5.09 (−5.92 to −4.26) | −4.89 (−5.86 to −4.21) |

AAMR = age adjusted mortality rate; APC = annual percent change; AAPC = average annual percent change. Both definitions were combined with cerebrovascular disease (I60 to I69). Sensitivity analysis rates are available to two decimal places and percent changes for that analysis were calculated from them. Annual data are in Supplemental Table 10 and full joinpoint estimates in Supplemental Table 11.
